# The Association of miR-29a and miR-29c with Carotid Intima–Media Thickness and Coronary Artery Disease Severity

**DOI:** 10.3390/genes17090987

**Published:** 2026-08-24

**Authors:** Mehmet Semih Belpinar, Hidayet Demir, Mehmet Altuğ Tunçer, Mehrdad Sheikhvatan

**Affiliations:** 1 Memorial Göztepe Hospital, Istanbul 34634, Turkey; 2 Department of Medical Genetics, Tehran University of Medical Sciences, Tehran 14176, Iran; 3 Department of Medical Biology and Genetics, Istanbul Okan University, Istanbul 34959, Turkey

**Keywords:** microRNA-29, coronary artery disease, carotid intima–media thickness

## Abstract

Background/Objectives: Carotid intima–media thickness (CIMT) is widely recognized as an established biomarker of systemic atherosclerosis and coronary artery disease (CAD). Nevertheless, the connection between circulating miR-29 family members, CIMT and CAD severity has not yet been determined. The aim of this study was to investigate the association of plasma miR-29a and miR-29c with CIMT and CAD severity. Methods: A total of 628 patients scheduled for elective coronary angiography were included in the study. CAD severity was estimated using the Gensini scoring system and was classified into mild, moderate, and severe categories. Additionally, CIMT was evaluated using high-resolution B-mode ultrasonography, while plasma miR-29a and miR-29c levels were detected using qRT-PCR. Results: CIMT increased significantly with CAD severity (0.76 ± 0.15, 0.94 ± 0.18, and 1.12 ± 0.22 mm for mild, moderate, and severe CAD, respectively; *p* < 0.001). MiR-29a expression progressively increased, whereas miR-29c expression decreased with advancing CAD (both *p* < 0.001). MiR-29a correlated positively with CIMT (r = 0.612) and Gensini score (r = 0.658), while miR-29c showed negative correlations (r = −0.527 and −0.584, respectively; all *p* < 0.001). CIMT, miR-29a, and miR-29c were found to be independently associated with CAD severity. Their combined model demonstrated a powerful association with severe CAD (AUC = 0.927; sensitivity 88.6%; specificity 84.1%). Conclusions: Plasma miR-29a and miR-29c are independent biomarkers of CIMT and CAD severity. Their combination with CIMT can improve diagnosis of severe CAD and may enhance cardiovascular risk stratification.

## 1. Introduction

Despite remarkable progress made in preventive strategies, diagnostic measures, and therapies, coronary artery disease (CAD) still holds the title of the number one cause of morbidity and mortality across the globe. Atherosclerosis, which is the main pathophysiological basis of CAD, is a progressive and chronic inflammatory/fibrotic disorder that consists of several stages: endothelial dysfunction, Vascular smooth muscle cells (VSMCs) remodeling, the extracellular matrix (ECM) changes, and plaque formation [1,2]. Since atherosclerosis is systemic in nature, vascular changes noted in carotid arteries usually correlate with the burden of coronary atherosclerosis [3]. Measurement of carotid intima–media thickness (CIMT) through ultrasonography became popular as a reliable surrogate marker of subclinical atherosclerosis and cardiovascular risk [4]. There are studies demonstrating statistically significant associations between the increased value of CIMT and the presence/severity of CAD [5,6].

While CIMT is a source of structural information on vascular remodeling, it is not able to provide insight into the complexity of molecular pathways responsible for the development of atherosclerosis [7]. More and more research is being conducted on circulating biomarkers that could serve as a reflection of biological processes and increase the accuracy of stratifying patients. One of these markers is microRNA (miRNA), which plays an important role in regulating gene expression and cardiovascular diseases [8]. It influences different pathways responsible for endothelial function, inflammation, lipids metabolism, fibrosis, and VSMC biology [9,10].

The miR-29 family comprises three major members, miR-29a, miR-29b, and miR-29c, which share considerable sequence homology and participate in the regulation of extracellular matrix (ECM) homeostasis. Among these members, miR-29b has been particularly investigated in vascular remodeling and fibrosis. It regulates the expression of several ECM components, including collagens, fibrillin-1, and elastin, and has been implicated in vascular smooth muscle cell proliferation and migration [11]. Experimental and clinical studies have also suggested that reduced miR-29b expression may contribute to ECM accumulation and vascular fibrosis, whereas altered miR-29b expression has been associated with atherosclerotic plaque remodeling and coronary atherosclerosis [11,12]. These observations indicate that miR-29b may have an important role in maintaining vascular wall integrity and modulating atherosclerotic processes.

Although the three members belong to the same family and share several biological targets, their circulating expression patterns and associations with different manifestations of vascular disease may not be identical. Previous evidence has identified miR-29b as a candidate circulating biomarker of coronary atherosclerosis, while miR-29a and miR-29c have also been associated with carotid atherosclerosis and vascular remodeling [12,13].

Therefore, investigating individual members of the miR-29 family may provide complementary information regarding different aspects of atherosclerotic disease. Studies have shown that miR-29a is capable of regulating the proliferation, migration, and invasion of VSMCs by affecting pro-inflammatory pathways [12,13].

Additional research studies have confirmed the correlation of plasma miR-29a levels with atherosclerotic burden [14]. The high levels of plasma miR-29a were observed to be positively correlated with CIMT and the presence of atherosclerosis; hence, miR-29a could act as a marker for vascular damage and subclinical atherosclerosis [14]. Furthermore, strong associations of miR-29a with carotid artery thickening reveal a connection between molecular alterations and structural vascular changes [15].

Conversely, the exact functions of miR-29c in the process of human atherosclerosis still remain to be elucidated. The data currently available indicate that miR-29c is involved in ECM turnover, fibrosis, and inflammation, which are all processes associated with the development of atherosclerotic plaques and intimal thickening [16]. Nevertheless, not many research papers have been devoted to the study of the correlation between the level of miR-29c in the bloodstream and CIMT or coronary atherosclerotic burden [17].

Considering the known correlation of CIMT and CAD severity and the role of miR-29 genes in recent studies of vascular biology, examination of these biomarkers might facilitate patient screening for coronary heart disease. Accordingly, the current study was conducted to examine the relationship between plasma miR-29a and miR-29c expression, CIMT and their significance in assessing the severity of CAD in patients evaluated via coronary angiography. Our hypothesis was that dysregulation of miR-29a and miR-29c might correlate with carotid arterial thickness and CAD severity, respectively.

## 2. Materials and Methods

### 2.1. Study Design and Patient Population

This cross-sectional study was performed in the department of cardiology of Sina Hospital in Tehran from 2022 to 2024. The participants recruited for this study consisted of consecutive patients undergoing elective coronary angiography due to CAD. Patients that met the inclusion criteria were defined as adults of at least 18 years old who had undergone carotid ultrasonography and coronary angiography during the study duration. Exclusion criteria included having a history of acute myocardial infarction in the last three months, infection, chronic inflammation, autoimmune disease, cancer, liver dysfunction, kidney disease, carotid surgery in the past, and immunosuppressive treatment. This 3-month window was chosen because the acute phase of myocardial infarction is known to induce a transient, self-limited perturbation of circulating and cellular microRNA profiles superimposed on the chronic atherosclerotic burden; prior work has shown that several cardiovascular-associated microRNAs remain altered for up to approximately 3 months after the index event before returning toward baseline [18]. Excluding only recent (3-month or shorter) infarction, rather than any history of myocardial infarction, was therefore intended to avoid confounding the assessment of miR-29a/miR-29c as markers of chronic, stable atherosclerotic burden with the transient miRNA perturbation of the acute post-infarction period, while preserving the generalizability of the cohort, since most patients referred for elective angiography have some prior cardiovascular history.

Age, gender, body mass index (BMI), smoking habit, hypertension, diabetes mellitus, dyslipidemia, family history of coronary artery disease, and medications were collected from each patient’s medical files and patient interviews. Blood pressure readings were collected based on clinical standards [19]. Blood pressure was measured by trained clinical personnel using a validated upper-arm blood pressure device and an appropriately sized cuff. Before measurement, participants were asked to rest quietly in a seated position for at least 5 min, with the back supported, feet flat on the floor, and legs uncrossed. The arm was supported at heart level, and the cuff was placed directly on the bare upper arm. Participants were instructed to avoid smoking, caffeine intake, and physical exercise for at least 30 min before measurement and to remain silent during the measurement procedure. At least two blood pressure readings were obtained, with measurements separated by approximately 1 min, and the average of the readings was used for analysis. Hypertension was defined as a previously established physician diagnosis of hypertension, current use of antihypertensive medication, or blood pressure ≥ 140/90 mmHg measured according to standard clinical procedures. Blood pressure was measured after the participant had been seated and resting, with an appropriately sized cuff. When multiple measurements were available, the mean value was used for analysis. Diabetes mellitus was defined as a previous physician diagnosis of diabetes mellitus, current use of glucose-lowering medication, fasting plasma glucose ≥ 126 mg/dL (7.0 mmol/L), or glycated hemoglobin (HbA1c) ≥ 6.5%. A random plasma glucose ≥ 200 mg/dL (11.1 mmol/L) in the presence of characteristic symptoms was also considered diagnostic. Dyslipidemia was defined as a previous physician diagnosis of dyslipidemia, current use of lipid-lowering medication, or the presence of an abnormal lipid profile. For the purposes of this study, abnormal lipid parameters were defined as total cholesterol ≥ 200 mg/dL, LDL cholesterol ≥ 130 mg/dL, triglycerides ≥ 150 mg/dL, or HDL cholesterol < 40 mg/dL in men or <50 mg/dL in women. Smoking status was obtained via direct patient interview and categorized as current smoker, former smoker, or never smoker. A current smoker was defined as a participant who reported active cigarette smoking at the time of study enrollment. A former smoker was defined as a participant who had previously smoked but had stopped before enrollment. Participants who had never regularly smoked were classified as never smokers. When available, smoking exposure was additionally recorded in pack-years, calculated as the average number of cigarette packs smoked per day multiplied by the number of years of smoking. Medication use was determined from the patient’s medical records and confirmed by patient interview. Medications were recorded by therapeutic class, including antihypertensive agents, antidiabetic medications, lipid-lowering agents (particularly statins), antiplatelet agents, anticoagulants, and other cardiovascular medications. Medication use was considered present when the participant was actively receiving the respective medication at the time of study assessment.

The project received clearance from the Institutional Ethics Committee of Tehran University of Medical Sciences (Approval No. IR.KMU.REC.1403.597). Informed consent was also obtained from each participant. This study was performed in compliance with the Helsinki declaration.

### 2.2. Clinical and Laboratory Assessments

Venous blood samples were obtained in the morning after an overnight fast of at least 8 h. Participants were instructed to avoid caloric intake during the fasting period, while water was permitted. The routine laboratory tests included fasting blood sugar, glycated hemoglobin (HbA1C), total cholesterol, triglyceride, LDL cholesterol, HDL cholesterol, serum creatinine, high sensitivity C-reactive protein (hs-CRP), and complete blood count. The biochemical tests were analyzed using automated systems in the central laboratory of the hospital as per instructions of the manufacturers. Fasting blood sugar and lipid profile (total cholesterol, triglycerides, LDL-C, HDL-C) were measured using enzymatic colorimetric method on a Roche Cobas c501/c701 autoanalyzer (Roche Diagnostics, Copenhagen, Denmark). HbA1c was determined by high-performance liquid chromatography (HPLC) using a Bio-Rad D-10 analyzer (Hercules, CA, USA). Serum creatinine was measured by the Jaffe method. hs-CRP was measured using immunoturbidimetric assay. Complete blood count was performed using a hematology analyzer (Mindray BC-5150, Shenzhen, China). All biochemical and hematological parameters were determined in a double measurement with the mean value used for analysis for each sample.

### 2.3. Coronary Angiography and Assessment of CAD Severity

Selective coronary angiography was performed following the standard Judkins technique via radial or femoral access. Coronary angiographic data were analyzed independently by two interventional cardiologists blinded to the microRNA and ultrasonography findings. In cases of disagreement regarding the degree of stenosis or Gensini score, consensus was reached through joint review and discussion; a third senior interventional cardiologist adjudicated the discrepancy so that the mean of the two readings was used. Inter-observer agreement between the two readers was assessed using kappa statistics. CAD was defined as significant when there was an obstruction of ≥50% in one of the major coronary arteries [20]. The severity of the disease was quantitatively assessed by Gensini scores that take into consideration the extent of luminal obstruction and the coronary segment anatomy. The Gensini score was calculated by assigning a severity score to each coronary segment based on the percentage of luminal diameter stenosis (1 point for ≤25% stenosis, 2 points for 26–50%, 4 points for 51–75%, 8 points for 76–90%, 16 points for 91–99%, and 32 points for total occlusion). Each segment score was then multiplied by a weighting factor reflecting the functional and anatomical importance of that coronary segment (e.g., a factor of 5 for the proximal left anterior descending artery and proximal circumflex artery, 2.5 for the mid left anterior descending artery, and lower factors for distal or less critical segments). The sum of the weighted scores for all diseased segments yielded the total Gensini score for each patient. Severity of CAD was classified as mild, moderate, and severe based on the Gensini score tertile distribution, corresponding to Gensini scores of 0–20 for mild, 21–40 for moderate, and >40 for severe [21]. Also, coronary vessel involvement was classified as single, two, or three-vessel disease; single-vessel disease was defined as significant stenosis in one major coronary artery, two-vessel disease as significant stenosis in two major coronary arteries, and three-vessel disease as significant stenosis in all three major coronary arteries.

### 2.4. Carotid Ultrasonography and CIMT Measurement

Carotid intima–media thickness (CIMT) was measured using a high-resolution B-mode ultrasound device using a linear array transducer with a frequency range of 7–12 MHz (Toshiba/Canon—Aplio series, linear transducer, PLT-1204BX, Otawara, Tochigi, Japan). All CIMT examinations were performed by a single specialized vascular sonographer who was blinded to the clinical and angiographic findings, in order to eliminate inter-observer variability. Intra-observer reproducibility was assessed by repeat measurements in a subset of X patients, showing an intraclass correlation coefficient of 0.88. The participants were scanned in the supine position with the neck extended and the head rotated to the contralateral side. Measurement was made at the distal common carotid artery from the far wall, approximately 1 cm proximal to the bifurcation. Three measurements were taken at end-diastole on each side, and the mean was calculated for data analysis. Mean CIMT was determined by averaging six measurements from both carotid arteries. Carotid plaque was identified as a focal structure bulging into the arterial lumen with a thickness greater than 1.5 mm or 50% larger than that of the surrounding vessel wall, in accordance with the Mannheim Carotid Intima–Media Thickness and Plaque Consensus [22].

### 2.5. Blood Collection and Plasma Preparation for microRNA Analysis

Blood drawn from peripheral veins (5 mL) using EDTA-coated syringes was collected prior to undergoing angiography of the coronary artery. Samples were kept at room temperature/4 °C and processed within exactly 2 h of collection to minimize RNA degradation and hemolysis/prevent pre-analytical variability in circulating miRNA levels. All samples across the cohort were processed within this same time window to ensure consistency of pre-analytical handling. The blood samples were then centrifuged at 3000× *g* for 15 min at 4 °C in order to isolate the plasma portion of the sample. All samples were subjected to the same centrifugation, plasma separation, aliquoting, and storage conditions. Samples were thawed only once immediately before RNA extraction and were processed using the same RNA extraction and qRT-PCR protocols.

### 2.6. RNA Extraction

Total RNA, which is highly enriched with small RNAs, was isolated from 200 μL of plasma with the miRNeasy Serum/Plasma Kit (Qiagen, Hilden, Germany) according to the manufacturer’s protocol. RNA quantity and quality were assessed via NanoDrop spectrophotometry (Thermo Fisher Scientific, Wilmington, DE, USA). Ratios of A260/A280 in the range 1.8–2.1 were regarded as satisfactory.

### 2.7. Reverse Transcription

The synthesis of cDNA was carried out using the TaqMan™ Advanced miRNA cDNA Synthesis Kit (Applied Biosystems, Foster City, CA, USA). Reverse transcription reactions were conducted in a reaction volume of 15 µL following the protocol recommended by the manufacturer. Reaction parameters involved polyadenylation, adapter ligation, reverse transcription, and miR amplification processes. cDNA was kept frozen at −20 °C until quantitative analysis.

### 2.8. Quantitative Real-Time Polymerase Chain Reaction (qRT-PCR)

The expression levels of miR-29a and miR-29c were quantified using pre-designed TaqMan™ MicroRNA Assays (Applied Biosystems, Thermo Fisher Scientific, Waltham, MA, USA) on a QuantStudio™ 5 Real-Time PCR System. The assay for human miR-29a-3p was Assay ID 002112 (miRBase accession number MIMAT0000086), and the assay for human miR-29c-3p was Assay ID 000587 (miRBase accession number MIMAT0000681). The mature miRNA sequences targeted by these assays were UAGCACCAUCUGAAAUCGGUUA (miR-29a-3p) and UAGCACCAUUUGAAAUCGGUUA (miR-29c-3p), respectively. Reverse transcription and quantitative real-time PCR were performed according to the manufacturer’s instructions.

### 2.9. miRNA Normalization and Relative Expression Analysis

To ensure consistency in miRNA quantification, both endogenous and exogenous normalization controls were incorporated into the analysis. Synthetic *Caenorhabditis elegans* cel-miR-39 was used as an exogenous spike-in control to monitor variation associated with RNA extraction and downstream processing. Briefly, 5 μL of cel-miR-39 spike-in control (1.6 × 10^8^ copies/μL) was added to each plasma sample after addition of QIAzol lysis reagent and before RNA extraction. The same amount of spike-in control was added to all samples. U6 small nuclear RNA (U6 snRNA) was used as the endogenous reference control. U6 was selected because it was consistently detectable across the analyzed plasma samples and showed stable amplification across the study samples, thereby providing a common reference for technical normalization of the qRT-PCR measurements. Because endogenous reference stability is an important consideration in circulating miRNA studies, U6-based normalization was complemented by the use of the exogenous cel-miR-39 spike-in control. The concordance of the normalization controls supported the consistency of the miRNA measurements across samples. For each sample, the Ct values of miR-29a and miR-29c were normalized to U6 using the comparative cycle threshold method. The ΔCt was calculated as follows:

ΔCt = Ct(miR-29a or miR-29c) − Ct(U6)

ΔΔCt was subsequently calculated relative to the mean ΔCt of the mild CAD reference group:

ΔΔCt = ΔCt(sample) − ΔCt(mild-CAD calibrator)

Relative miRNA expression was calculated using the comparative method:

Relative expression = 2^−ΔΔCt^

The mild CAD group was assigned a relative expression value of 1.00 and served as the calibrator for comparisons with the moderate and severe CAD groups. This normalization procedure generated the fold-change values reported for miR-29a and miR-29c in Table 1.

### 2.10. Statistical Analysis

All statistical tests were conducted using the Statistical Package for Social Sciences software package 27.0 (SPSS, Inc., Chicago, IL, USA) and GraphPad Prism 10.0. Continuous variables were checked for normal distribution by using the Shapiro–Wilk test. The normally distributed continuous variables were reported as mean ± standard deviation (SD). In case of failure in normal distribution, non-normally distributed continuous variables were reported as the median (interquartile range). The categorical variables were reported as frequencies and percentages. Independent-samples *t*-test or one-way analysis of variance (ANOVA) was used for the comparison between groups according to their distribution characteristics. The Mann–Whitney U test or Kruskal–Wallis test was applied for comparison of non-parametric variables. For categorical variables, the χ^2^ test or Fisher’s exact test was used. For evaluating correlations between expression level of microRNAs, CIMT measurement and Gensini score, Pearson or Spearman correlation test was conducted. Multivariate linear regression model was conducted to reveal independent predictors of CIMT and CAD severity adjusted for traditional risk factors for cardiovascular disease. Receiver operating characteristic (ROC) curve analysis was performed to assess the diagnostic performance of miR-29a, miR-29c, and CIMT in relation to severe CAD. *p* value < 0.05 was regarded as statistically significant.

## 3. Results

### 3.1. Study Population and Baseline Characteristics

**Figure 1:**
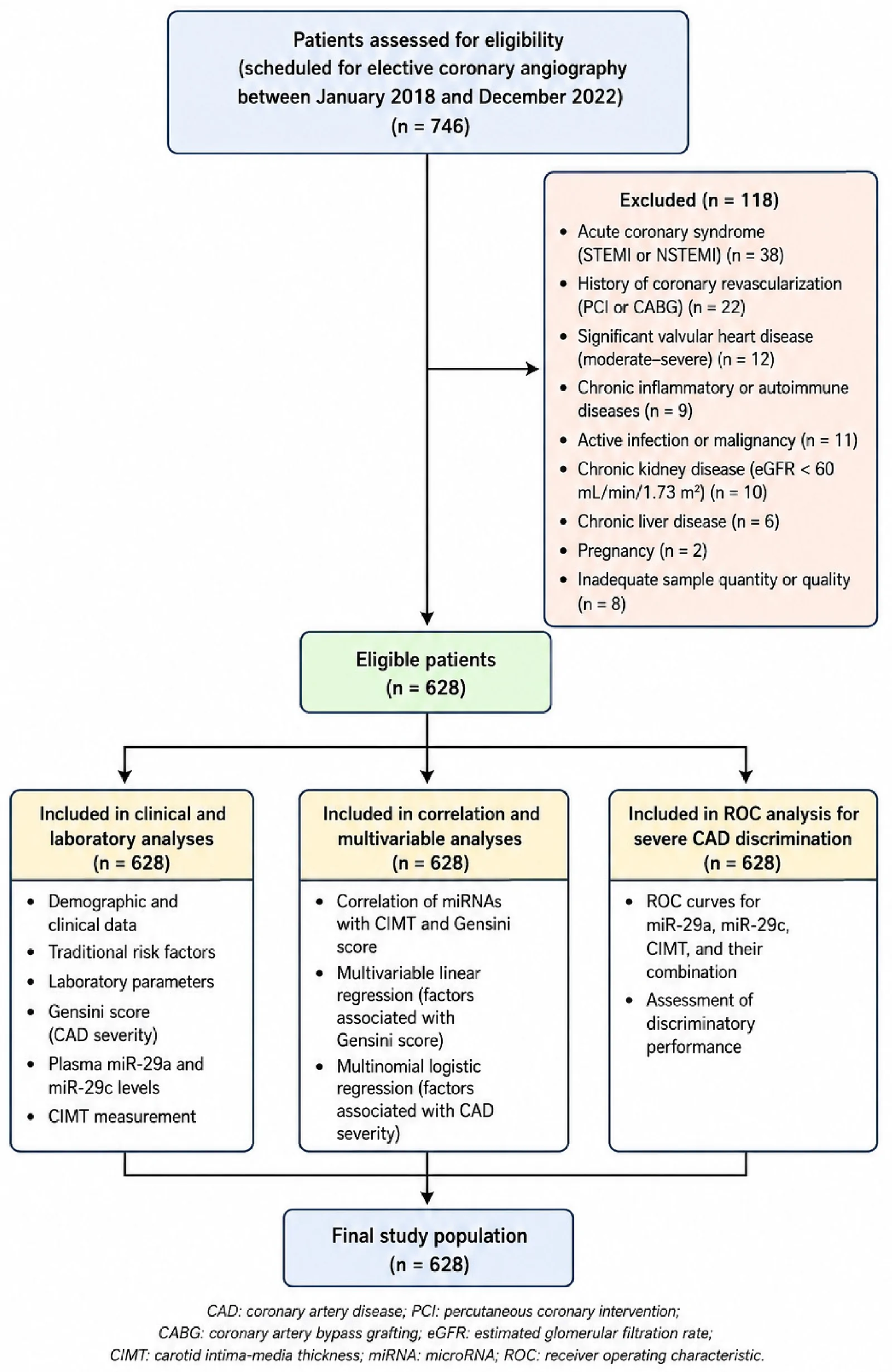
The study population flowchart showing the eligibility assessment, inclusion/exclusion criteria, and final cohort of 628 participants.

A total of 628 patients who underwent elective coronary angiography were recruited in the present study (Figure 1). The mean age of the patients in the study was 62.4 ± 10.8 years, and males accounted for 402 (64.0%). Patients were classified as having mild CAD (n = 209), moderate CAD (n = 209), and severe CAD (n = 210) according to coronary angiogram and Gensini score tertiles.

The baseline demographic, clinical, and laboratory data of the studied patients are listed in Table 2. Patients with severe CAD were found to be significantly older than those with mild CAD (66.1 ± 10.7 vs. 58.7 ± 9.6 years; *p* < 0.001). The prevalence of classical risk factors for cardiovascular disease increased progressively with increasing severity of CAD. The incidence of hypertension was significantly higher among patients with severe CAD (73.8%) as compared to those with mild CAD (49.8%; *p* < 0.001). Moreover, diabetes mellitus was identified in 52.4% and 28.2% of patients with severe and mild CAD, respectively (*p* < 0.001). Likewise, the prevalence of smoking and high plasma levels of LDL cholesterol were significantly higher in patients with severe CAD. Also, systemic inflammation was significantly higher among the more severely ill group, as indicated by hs-CRP levels. The levels of hs-CRP were found to be elevated two folds in patients with severe CAD as compared to mild CAD (5.8 ± 2.9 vs. 2.8 ± 1.6 mg/L, *p* < 0.001).

### 3.2. Carotid Intima–Media Thickness According to CAD Severity

**Figure 2:**
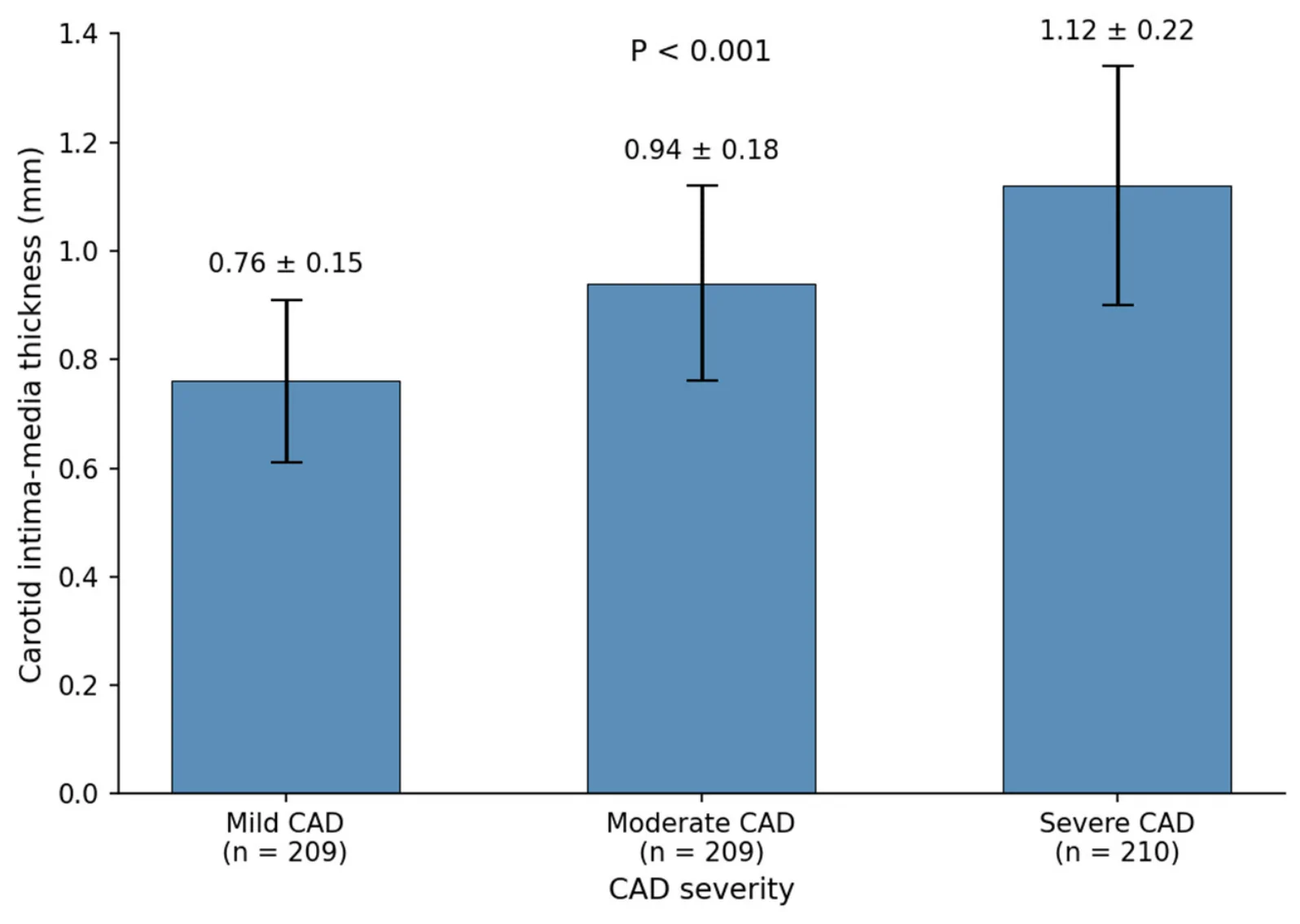
The distribution of carotid intima–media thickness (CIMT) according to CAD severity. Individual data points are shown for all participants, with boxplots illustrating the median, interquartile range, and the distribution of CIMT values in the mild, moderate, and severe CAD groups.

The mean CIMT varied significantly between the three CAD severity groups (Table 1). Subjects with severe CAD had highly elevated levels of CIMT values compared to those with moderate and mild CAD (1.12 ± 0.22 mm, 0.94 ± 0.18 mm, and 0.76 ± 0.15 mm, respectively; *p* < 0.001). There was a gradual increment in the value of CIMT in all the severity grades of coronary disease. This showed that there was a strong connection between the burden of coronary and carotid atherosclerosis. In addition, the frequency of carotid plaques rose significantly from 24.9% in the mild CAD category to 68.1% in the severe CAD category (*p* < 0.001). As shown in Figure 2, the CIMT readings exhibited a stepwise increase from mild to severe CAD with minimal overlapping between the groups. Post hoc testing revealed that there were significant differences between all pairwise comparisons (all adjusted *p* < 0.05).

### 3.3. Plasma Expression Levels of miR-29a and miR-29c

**Figure 3:**
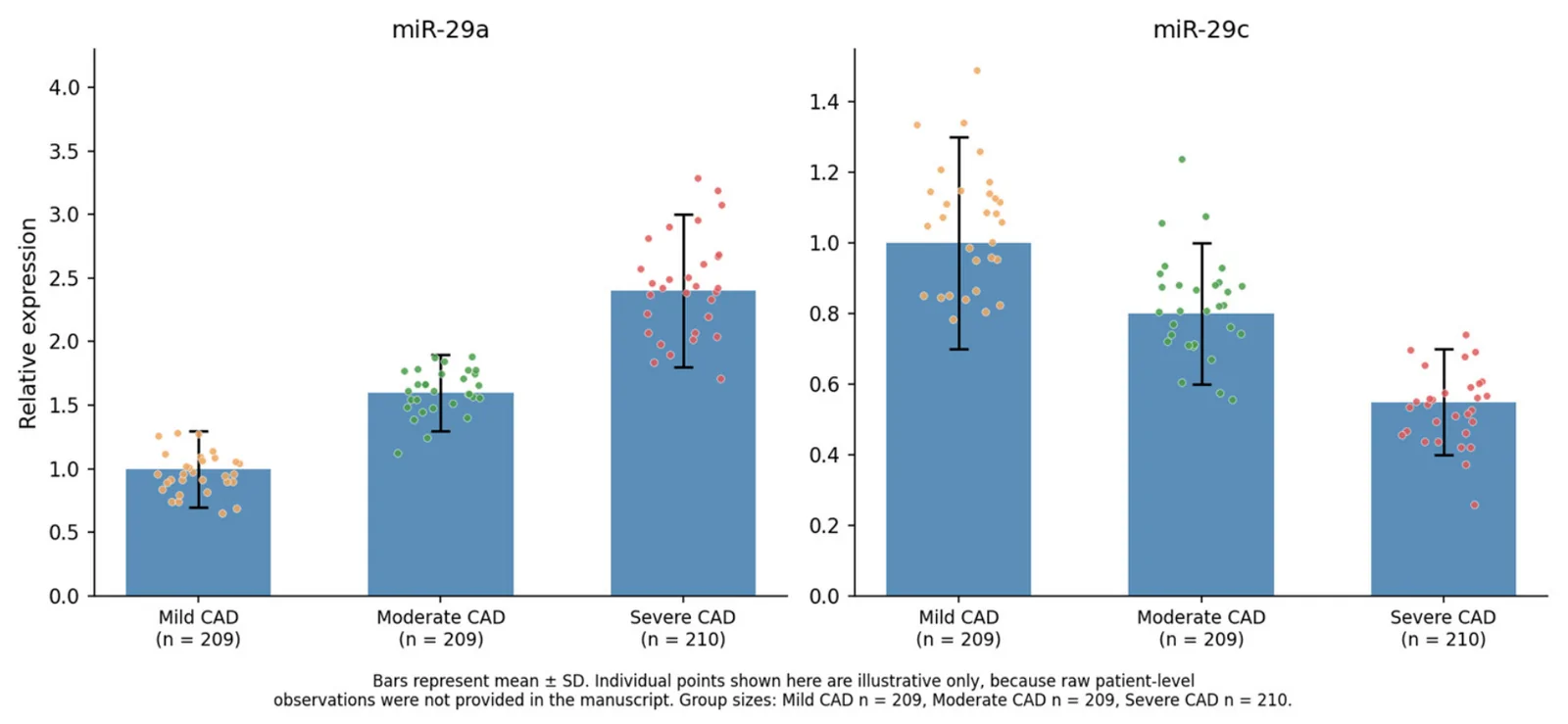
Relative expression levels of miR-29a and miR-29c in mild, moderate, and severe CAD groups. Bars represent mean ± SD, with individual data points overlaid to demonstrate distribution of observations.

Plasma expression levels of miR-29a and miR-29c are depicted in Table 1 and Figure 3. Plasma levels of miR-29a were found to be significantly elevated in patients suffering from advanced forms of CAD. The average relative expression rose from 1.00 ± 0.31 in the mild CAD group to 1.62 ± 0.48 in the moderate CAD group and further rose to 2.43 ± 0.71 in the severe CAD group (*p* < 0.001). On the other hand, plasma levels of miR-29c were observed to fall with increasing CAD severity. There was a decrease in relative expression from 1.00 ± 0.27 in the mild CAD group to 0.79 ± 0.22 in the moderate CAD group and then further to 0.56 ± 0.18 in the severe CAD group (*p* < 0.001). As shown in Figure 3, miR-29a and miR-29c have contrasting expression profiles, with the former being gradually upregulated while the latter is markedly downregulated.

### 3.4. Correlation Between microRNA Expression and Carotid Intima–Media Thickness

**Figure 4:**
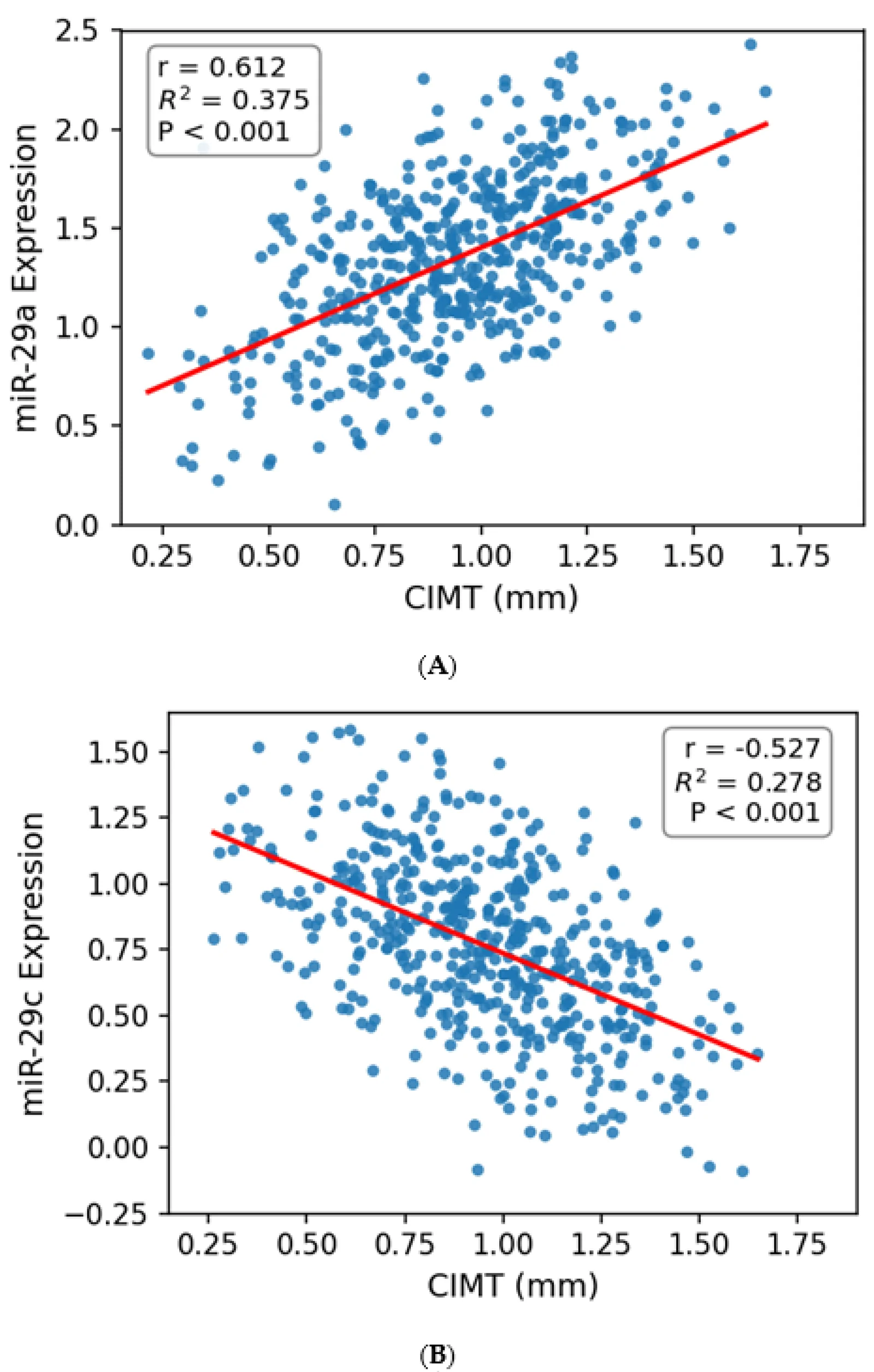

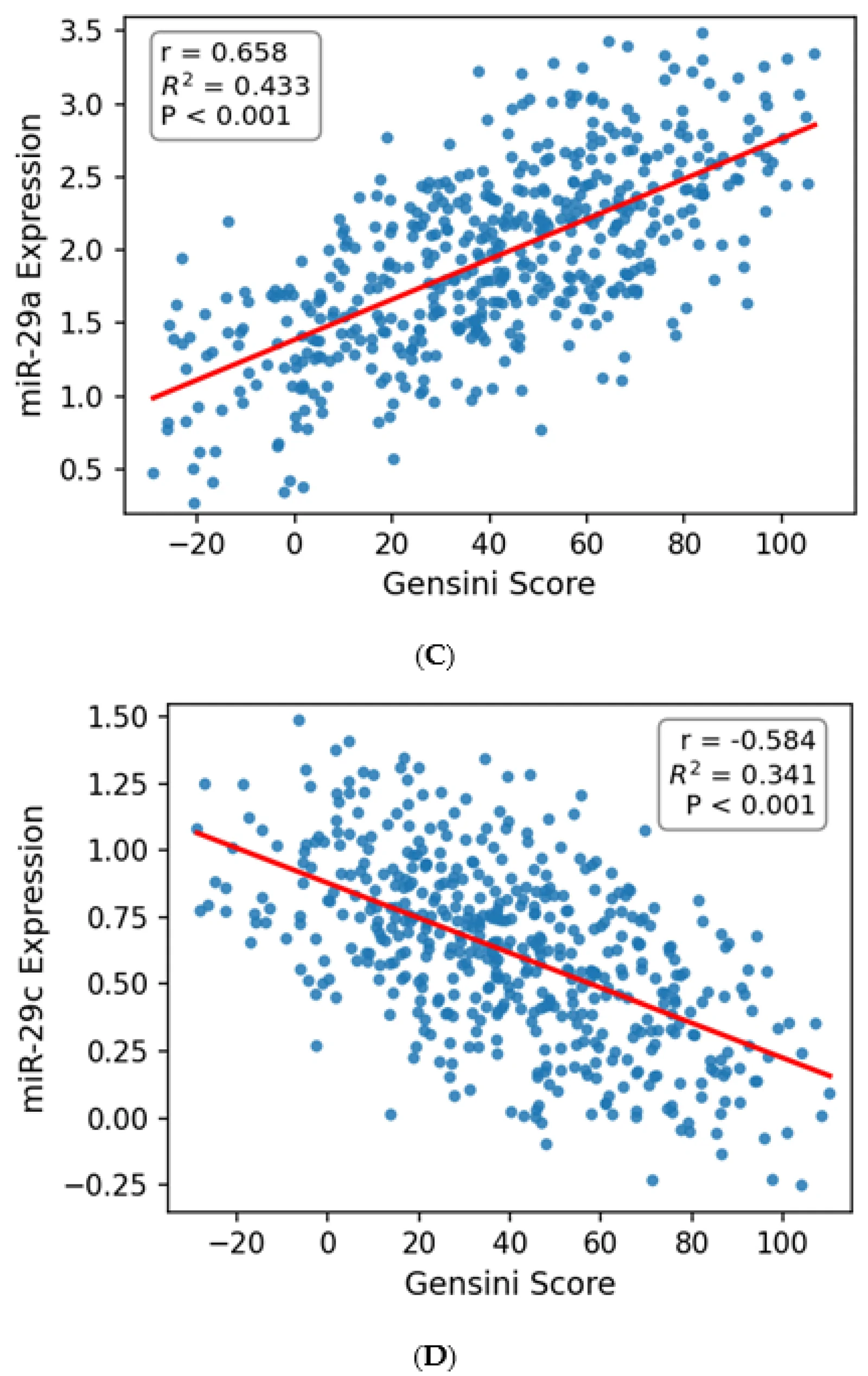
Correlation of miR-29a with CIMT (**A**), miR-29c with CIMT (**B**), miR-29a with Gensini score (**C**), and miR-29c with Gensini score (**D**).

There was a significant correlation found between the levels of microRNAs and CIMT values. The miR-29a level showed significant positive correlation with CIMT (r = 0.612, *p* < 0.001), showing that higher levels of miR-29a in the plasma had an increased CIMT value, suggesting that there was increased thickness of the carotid artery walls (Figure 4A). On the other hand, miR-29c levels exhibited a significant negative correlation with CIMT (r = −0.527, *p* < 0.001), implying that lower levels of miR-29c corresponded to high CIMT values, suggesting that there is an increase in atherosclerosis in carotid arteries (Figure 4B).

### 3.5. Correlation Between microRNA Expression and Coronary Disease Severity

Moreover, the association between circulating microRNAs and coronary atherosclerotic burden was confirmed by further analyses. In particular, miR-29a showed a high level of positive correlation with the Gensini score (r = 0.658, *p* < 0.001), while miR-29c had an inverse correlation (r = −0.584, *p* < 0.001). Correlations between the analyzed variables are presented graphically in Figure 4C,D. Patients who showed the highest Gensini scores always had increased miR-29a concentrations and decreased levels of miR-29c. At the same time, correlations with serum hs-CRP concentrations were observed for both microRNAs. Specifically, there was a positive correlation between miR-29a and hs-CRP (r = 0.419, *p* < 0.001) and an inverse correlation between miR-29c and hs-CRP (r = −0.332, *p* < 0.001).

### 3.6. Relationship Between Biomarkers and Extent of Coronary Vessel Involvement

The relationship between biomarker expression and angiographic extent of CAD is shown in Table 3. Patients with three-vessel disease exhibited the highest miR-29a levels (2.57 ± 0.69-fold change) and the lowest miR-29c levels (0.49 ± 0.16-fold change). In contrast, patients with single-vessel disease showed significantly lower miR-29a expression (1.31 ± 0.44) and higher miR-29c expression (0.91 ± 0.25) (both *p* < 0.001). Similarly, CIMT increased significantly with the extent of coronary involvement, reaching 1.18 ± 0.23 mm in patients with three-vessel disease compared with 0.82 ± 0.17 mm among patients with single-vessel disease (*p* < 0.001). These findings further support the association between carotid vascular remodeling, microRNA dysregulation, and the overall burden of coronary atherosclerosis.

### 3.7. Multivariable Analysis of Predictors of CAD Severity

In order to see if miR-29a and miR-29c were closely associated with CAD severity, multivariate linear regression analysis was performed after controlling for the usual cardiovascular risk factors (Table 4). Age, diabetes mellitus, hs-CRP levels, CIMT, miR-29a expression, and miR-29c expression turned out to be associated with Gensini score. Of all the tested variables, miR-29a turned out to be one of the most powerful determinants of CAD severity (β = 0.37, 95% CI: 0.25–0.49, *p* < 0.001). CIMT was also shown to have a considerable independent relationship with Gensini score (β = 0.31, 95% CI: 0.20–0.43, *p* < 0.001). On the other hand, miR-29c was significantly related to reduced CAD severity (β = −0.29, 95% CI: −0.40 to −0.17, *p* < 0.001).

### 3.8. Diagnostic Performance of miR-29a, miR-29c, and CIMT

**Figure 5:**
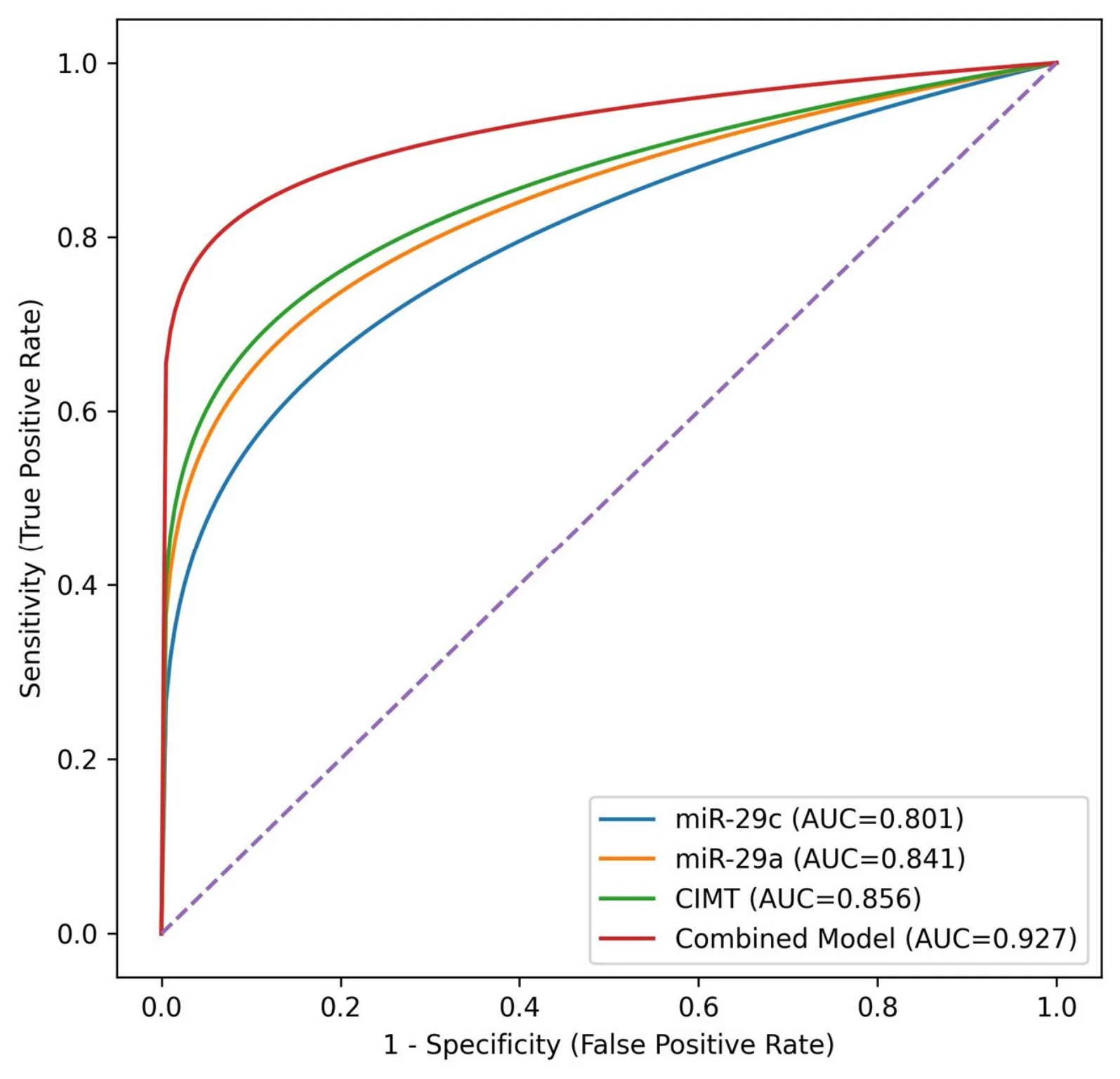
ROC curves for miR-29a, miR-29c, CIMT, and the combined predictive model.

Receiver operating characteristic (ROC) analyses were performed to evaluate the ability of the investigated biomarkers to identify severe CAD (Figure 5). Among the individual biomarkers, ROC analysis was carried out to determine the capacity of the tested biomarkers to discriminate between severe CAD (Figure 5). Among the single biomarkers, CIMT showed the highest accuracy with an area under the curve (AUC) of 0.856 (95% CI: 0.824–0.888). The AUCs of miR-29a and miR-29c were 0.841 (95% CI: 0.809–0.873) and 0.801 (95% CI: 0.764–0.838), respectively. Remarkably, the combined model consisting of miR-29a, miR-29c, and CIMT displayed improved discriminatory power, resulting in AUC of 0.927 (95% CI: 0.906–0.948). Using the best cut-off point, the combined model could discriminate severe CAD with 88.6% sensitivity and 84.1% specificity. As shown in Figure 5, the combined biomarker model significantly outperformed each individual marker, indicating substantial incremental value from integrating molecular and imaging biomarkers.

### 3.9. Subgroup Analyses

The subgroup analysis revealed that the identified associations were stable regardless of whether or not the patients had diabetes. The links among miR-29a, miR-29c, CIMT, and Gensini score were maintained within both subgroups and no interactions were found between having or not having diabetes and the ability of microRNAs to predict disease (*p*-value of interaction for all comparisons was above 0.05). This indicates that the identified associations were robust and diabetes-related.

### 3.10. Correlation of miRNA Levels with Baseline Parameters

Correlation analysis revealed that circulating miR-29a levels were significantly and positively associated with several clinical and biochemical parameters (Table 5), including age (r = 0.142, *p* = 0.003), BMI (r = 0.187, *p* < 0.001), diabetes mellitus (r = 0.203, *p* < 0.001), hypertension (r = 0.221, *p* < 0.001), current smoking (r = 0.134, *p* = 0.005), dyslipidemia (r = 0.156, *p* = 0.001), fasting glucose (r = 0.176, *p* < 0.001), HbA1c (r = 0.198, *p* < 0.001), total cholesterol (r = 0.121, *p* = 0.008), LDL-C (r = 0.145, *p* = 0.002), triglycerides (r = 0.133, *p* = 0.006), and hs-CRP (r = 0.245, *p* < 0.001). A significant inverse correlation was observed between miR-29a and HDL-C (r = −0.167, *p* = 0.001). Conversely, miR-29c levels were significantly and negatively correlated with age (r = −0.098, *p* = 0.041), BMI (r = −0.165, *p* = 0.001), diabetes mellitus (r = −0.178, *p* < 0.001), hypertension (r = −0.196, *p* < 0.001), current smoking (r = −0.121, *p* = 0.011), dyslipidemia (r = −0.142, *p* = 0.003), fasting glucose (r = −0.159, *p* = 0.001), HbA1c (r = −0.171, *p* < 0.001), total cholesterol (r = −0.110, *p* = 0.016), LDL-C (r = −0.132, *p* = 0.005), triglycerides (r = −0.119, *p* = 0.012), and hs-CRP (r = −0.219, *p* < 0.001), and positively correlated with HDL-C (r = 0.153, *p* = 0.001). No significant correlations were found between either miRNA and current pharmacological treatment, including statin use (miR-29a: r = −0.089, *p* = 0.062; miR-29c: r = 0.076, *p* = 0.104), antiplatelet therapy (r = −0.045, *p* = 0.310; r = 0.052, *p* = 0.247), or ACEi/ARB use (r = 0.062, *p* = 0.160; r = −0.058, *p* = 0.189), suggesting that these medications did not substantially confound miRNA expression levels in this cohort (Table 1).

Values represent Pearson correlation coefficients (r) for continuous variables and point-biserial correlation coefficients for binary variables (yes/no), relating each variable to circulating miR-29a and miR-29c expression levels. *p* < 0.05 was considered statistically significant (bold). BMI, body mass index; ACEi/ARB, angiotensin-converting enzyme inhibitor/angiotensin receptor blocker; HbA1c, glycated hemoglobin; LDL-C, low-density lipoprotein cholesterol; HDL-C, high-density lipoprotein cholesterol; hs-CRP, high-sensitivity C-reactive protein.

## 4. Discussion

The current study examined the association between circulating expression of miR-29a and miR-29c, CIMT, and the severity of CAD among a large population of individuals undergoing coronary angiography. The key results of the study are as follows: (1) CIMT progressively increased according to the severity of CAD; (2) circulating levels of miR-29a were found to be significantly higher in individuals with more severe coronary atherosclerosis, while levels of miR-29c were lower; (3) both miRNAs were independently associated with CIMT and the degree of coronary involvement; (4) miR-29a and miR-29c had significant associations with CAD severity even after correction for traditional cardiovascular risk factors; and (5) the combination of miR-29a, miR-29c, and CIMT showed high ability to discriminate severe CAD. Altogether, these data indicate the tight relation of miR-29 family members with the process of vascular remodeling.

The high correlation noted between CIMT and CAD has been found to be congruent with the theory that atherosclerosis is a systemic vascular disease involving simultaneous occurrence of disease process in various vascular beds. CIMT has long been considered an effective non-invasive tool for measuring the presence of subclinical atherosclerosis and has been linked to predict future cardiovascular outcomes. Earlier studies conducted by Lorenz et al. [23] and Den Ruijter et al. [24] have demonstrated the relationship between increased CIMT and the incidence of myocardial infarction and stroke, indicative of widespread atherosclerosis. Likewise, many studies using angiographic techniques have shown correlations between CIMT and the degree of coronary stenosis, indicating the effectiveness of carotid ultrasound in predicting coronary disease severity [25].

The results of this current study have shown an increasing trend of CIMT according to the increasing severity of CAD categories, and CIMT was found to be independently associated with the Gensini score. Also, it is noteworthy that patients with multi-vessel CAD have higher CIMT compared with those with mono-vessel disease. Such results confirm the already proven facts that carotid arterial remodeling is similar to coronary atherosclerotic development.

Another important finding from this study was the substantial increase in the concentration of circulating miR-29a in patients with advanced CAD. In addition, there were highly significant positive associations between miR-29a expression level and CIMT and Gensini score.

The miR-29 family has recently been implicated in the regulation of ECM turnover, fibrotic responses, inflammation, and VSMC functions [8,9]. The results of experimental investigations have indicated the role of miR-29a in modulating collagen biosynthesis, matrix metalloproteinases activities, and inflammation-related signaling processes involved in vessel remodeling. Their dysregulation can lead to atherosclerotic plaque instability and lesion development [26,27].

These results support those previously reported showing an increase in miR-29a concentration in the bloodstream of patients suffering from atherosclerosis. Huang et al. [28] found a significant correlation between the plasma concentration of miR-29a and carotid artery thickness, and Liu et al. [27] found a correlation between increased expression of miR-29a and increased CIMT and atherosclerotic load. Moreover, there have been experiments conducted showing that miR-29a can influence VSMC proliferation and migration through TNFRSF1A-induced inflammation [29].

On the other hand, blood miR-29c was found to be significantly decreased in CAD patients with more advanced disease and inversely associated with both CIMT and Gensini scores. Despite being a subject of fewer studies than miR-29a, emerging data indicate that miR-29c is involved in the modulation of ECM metabolism, fibrosis, and inflammatory processes [30]. A number of studies have shown that low expression levels of miR-29 family members result in increased deposition of collagen and remodeling of tissues. In the vascular wall, inhibition of miR-29c can lead to the creation of a pro-fibrotic environment conducive to formation of plaques and stiffness of arteries [8,9,10].

However, the inverse relationship found in our study between miR-29c expression and the severity of CAD has biological plausibility and indicates that there might be a potential protective effect of this microRNA. Low levels of miR-29c expression may lead to extracellular matrix accumulation and vascular fibrosis, which may result in progression of atherosclerosis. While the clinical significance is not well established yet, our results complement the existing literature on the topic.

One of the strong aspects of this study was the evaluation of both molecular and imaging biomarkers together. Whereas CIMT denotes the structural changes in arteries, circulating miRNAs offer valuable insights regarding biological mechanisms that take place in the walls of blood vessels. The model which integrates miR-29a, miR-29c and CIMT had an AUC value higher than 0.90, demonstrating better discrimination compared to other biomarkers alone.

It is worth noting that the idea of the use of circulating microRNAs with imaging markers is gaining popularity among researchers working in the field of cardiology. There are numerous studies that indicate the superiority of the multimarker approach compared to single biomarkers [31]. Our results confirm the effectiveness of such an approach, and we can suppose that its combination with CIMT evaluation can be promising in predicting advanced coronary disease.

These processes are likely to be complex and multicausal. Atherosclerosis includes chronic inflammation, endothelial dysfunction, lipid deposition, oxidative stress, and matrix remodeling. Members of the miR-29 family were reported to regulate genes responsible for collagen formation, elastin generation, TGF-β signaling, and inflammation [32]. Elevated levels of miR-29a could indicate the involvement of inflammation and matrix remodeling mechanisms, while low levels of miR-29c might promote excessive fibrosis and wall thickening due to lack of inhibition of extracellular matrix production [33]. Strong correlations with CIMT indicate a role of miR-29 family signaling in the pathogenesis of arterial remodeling. More detailed studies should be conducted to reveal the exact molecular mechanisms mediating the impact of the miR-29 family on coronary artery remodeling and coronary atherosclerosis.

An interesting finding of the present study was the divergent behavior of miR-29a and miR-29c despite their belonging to the same miR-29 family. Although these miRNAs share a highly conserved seed region and have overlapping target genes, individual miR-29 members can undergo differential transcriptional and post-transcriptional regulation, exhibit different intracellular distributions and stability, and respond differently to cellular and environmental stimuli. In CAD, this divergence may also reflect the complex cellular origin of circulating miRNAs. Circulating miRNA concentrations represent a composite contribution from endothelial cells, vascular smooth muscle cells, macrophages, platelets, and other circulating cells and may be influenced by differential secretion, extracellular-vesicle transport, cellular uptake, and degradation. Consequently, circulating miR-29a and miR-29c levels do not necessarily change in parallel or directly mirror their expression within atherosclerotic plaques. Moreover, miR-29 signaling appears to be highly context-dependent, with evidence suggesting both pro- and anti-atherogenic effects depending on the specific cell type and molecular environment. Therefore, the opposite directions observed for miR-29a and miR-29c in our cohort may represent distinct regulatory responses to CAD-related inflammatory, metabolic, and vascular processes. However, because our study was observational and measured circulating miRNAs at a single time point, this interpretation remains hypothetical and requires confirmation in mechanistic and longitudinal studies. Additionally, to further explore the potential biological relevance of these findings, we considered predicted gene targets of miR-29a-3p and miR-29c-3p using established miRNA target prediction resources. Because these miRNAs share a highly conserved seed region, considerable overlap in their predicted targets is expected. Several predicted targets are involved in extracellular matrix organization and vascular remodeling, including COL1A1, COL3A1, COL5A1, FBN1, and ELN, as well as matrix remodeling (MMP2 and MMP9) and epigenetic regulation (DNMT3A and DNMT3B). These pathways are biologically relevant to vascular wall remodeling and atherosclerotic plaque development and may provide potential mechanisms linking altered miR-29a/c expression with CAD severity. However, these interactions should be interpreted as predicted or previously reported targets rather than experimentally validated relationships in the present cohort, and further functional studies are required to confirm their relevance.

This study’s results can have several possible implications for clinical practice. First, the determination of levels of circulating miR-29a and miR-29c might give an opportunity to obtain extra data about the coronary atherosclerotic burden in the course of cardiological examination. Second, the combination of the results of microRNA profiling and CIMT assessment might help to better identify patients with advanced CAD using non-invasive methods. Third, the associations found indicate the possibility to use miR-29 family members as drug targets in the case of atherosclerotic vascular disease.

Several limitations need to be taken into account while interpreting the results obtained by this study. Firstly, the cross-sectional nature of this study does not allow us to establish any causality between microRNAs and the progression of the disease. Secondly, all patients included in this study were recruited from a single tertiary referral center; hence, the results might be limited in their generalizability. Thirdly, microRNAs were only assessed at one point of time and could not provide information regarding temporal changes during disease development. Fourth, circulating miRNAs were assessed at a single time point; therefore, the present cross-sectional design cannot determine whether miR-29a and miR-29c levels change over time or whether their changes parallel the progression or regression of coronary atherosclerosis. Longitudinal studies with serial plasma sampling and repeated assessment of coronary and carotid atherosclerotic burden are warranted to determine the temporal dynamics of these miRNAs and to clarify whether changes in miR-29a and miR-29c precede, accompany, or follow changes in atherosclerotic disease severity. Such studies are particularly important for determining the potential prognostic and monitoring value of these circulating biomarkers. Fifth, even though multivariable adjustment was performed, residual confounding is still possible. An additional limitation is that hemolysis was not quantitatively assessed in the collected plasma samples. Although blood samples were collected using standardized procedures and processed within 2 h to minimize pre-analytical variation, subclinical hemolysis could potentially influence circulating miRNA measurements. Therefore, a minor contribution of hemolysis to the observed miR-29a and miR-29c levels cannot be completely excluded. Future studies should incorporate a quantitative hemolysis assessment, such as spectrophotometric measurement of free hemoglobin and/or validated hemolysis-sensitive miRNA ratios, to further control for this potential source of bias. Also, mechanistic experiments were out of the scope of the current study and therefore any pathway through which miR-29 family members could exert their effect is speculative. Finally, although medication use was recorded by therapeutic class (including statins, antiplatelet agents, and anticoagulants) and considered a covariate, the specific statin compound, dose, and duration of use were not captured. This is a relevant limitation because statins have been reported to differentially and directly modulate expression of the miR-29 family independent of their lipid-lowering effect, with atorvastatin, but not simvastatin, repressing miR-29a-3p and miR-29b-3p in peripheral blood cells of hyperlipidemic subjects [34]. Because statin use was common in this CAD cohort and its prevalence is likely to have differed across CAD severity groups, residual confounding of the miR-29a/miR-29c associations by statin type cannot be excluded, and future studies should record and adjust for the specific statin agent and treatment duration.

## 5. Conclusions

In summary, this study showed a significant association between circulating miR-29a and miR-29c levels and carotid IMT and angiographically assessed coronary artery disease severity. The upregulation of miR-29a and downregulation of miR-29c were independently associated with the presence of more severe coronary atherosclerosis and vascular remodeling. In addition, the combined miR-29a, miR-29c, and CIMT assessment allowed us to achieve high discrimination power in severe CAD detection, better than using any single test. Therefore, our results indicate that a combination of molecular markers and vascular imaging can improve the evaluation of CAD severity and open up the possibility of using members of the miR-29 family as new biomarkers of atherosclerotic CVD.

## Figures and Tables

**Table 1:** Expression levels of miR-29a and miR-29c according to CAD severity.

Biomarker	Mild CAD	Moderate CAD	Severe CAD	*p*-Value
miR-29a (fold change)	1.00 ± 0.31	1.62 ± 0.48	2.43 ± 0.71	<0.001
miR-29c (fold change)	1.00 ± 0.27	0.79 ± 0.22	0.56 ± 0.18	<0.001
CIMT (mm)	0.76 ± 0.15	0.94 ± 0.18	1.12 ± 0.22	<0.001

**Table 2:** Baseline characteristics of study population.

Variable	Mild CAD (n = 209)	Moderate CAD (n = 209)	Severe CAD (n = 210)	*p*-Value
Age (years)	58.7 ± 9.6	62.3 ± 10.4	66.1 ± 10.7	<0.001
Male sex (%)	58.4	64.1	69.5	0.032
Hypertension (%)	49.8	61.2	73.8	<0.001
Diabetes mellitus (%)	28.2	39.7	52.4	<0.001
Current smoker (%)	26.8	35.4	44.3	0.001
LDL-C (mg/dL)	112 ± 34	118 ± 36	126 ± 39	0.004
hs-CRP (mg/L)	2.8 ± 1.6	4.1 ± 2.3	5.8 ± 2.9	<0.001

**Table 3:** Biomarker levels according to extent of coronary vessel involvement.

Variable	Single-Vessel (n = 230)	Two-Vessel (n = 208)	Three-Vessel (n = 190)	*p*-Value
miR-29a	1.31 ± 0.44	1.86 ± 0.53	2.57 ± 0.69	<0.001
miR-29c	0.91 ± 0.25	0.73 ± 0.22	0.49 ± 0.16	<0.001
CIMT (mm)	0.82 ± 0.17	0.99 ± 0.19	1.18 ± 0.23	<0.001

**Table 4:** Multivariable predictors of CAD severity (Gensini score).

Variable	β Coefficient	95% CI	*p*-Value
Age	0.21	0.11–0.33	0.001
Diabetes mellitus	0.18	0.08–0.27	0.004
hs-CRP	0.24	0.14–0.35	<0.001
CIMT	0.31	0.20–0.43	<0.001
miR-29a	0.37	0.25–0.49	<0.001
miR-29c	−0.29	−0.40 to −0.17	<0.001

**Table 5:** Correlation between miR-29a and miR-29c expression levels and clinical, demographic, and biochemical variables.

Variable	miR-29a r	*p* Value	miR-29c r	*p* Value
Age, years	0.142	0.003	−0.098	0.041
BMI, kg/m^2^	0.187	<0.001	−0.165	0.001
Diabetes mellitus, yes/no	0.203	<0.001	−0.178	<0.001
Hypertension, yes/no	0.221	<0.001	−0.196	<0.001
Current smoking, yes/no	0.134	0.005	−0.121	0.011
Dyslipidemia, yes/no	0.156	0.001	−0.142	0.003
Statin use, yes/no	−0.089	0.062	0.076	0.104
Antiplatelet therapy, yes/no	−0.045	0.310	0.052	0.247
ACEi/ARB use, yes/no	0.062	0.160	−0.058	0.189
Fasting glucose, mg/dL	0.176	<0.001	−0.159	0.001
HbA1c, %	0.198	<0.001	−0.171	<0.001
Total cholesterol, mg/dL	0.121	0.008	−0.110	0.016
LDL-C, mg/dL	0.145	0.002	−0.132	0.005
HDL-C, mg/dL	−0.167	0.001	0.153	0.001
Triglycerides, mg/dL	0.133	0.006	−0.119	0.012
hs-CRP, mg/L	0.245	<0.001	−0.219	<0.001

## Data Availability

The data that support the findings of this study are available from the corresponding author upon reasonable request.
